# Vector competence of *Haemaphysalis longicornis ticks* for a Japanese isolate of the Thogoto virus

**DOI:** 10.1038/s41598-018-27483-1

**Published:** 2018-06-18

**Authors:** Melbourne Rio Talactac, Kentaro Yoshii, Emmanuel Pacia Hernandez, Kodai Kusakisako, Remil Linggatong Galay, Kozo Fujisaki, Masami Mochizuki, Tetsuya Tanaka

**Affiliations:** 1 Laboratory of Infectious Diseases, Joint Faculty of Veterinary Medicine, Kagoshima University, 1-21-24 Korimoto, Kagoshima, 890-0065 Japan; 2 Department of Pathological and Preventive Veterinary Science, The United Graduate School of Veterinary Science, Yamaguchi University, Yoshida, Yamaguchi, 753-8515 Japan; 3 Laboratory of Public Health, Faculty of Veterinary Medicine, Hokkaido University, Kita-ku Kita-18 Nishi-9, Sapporo, Hokkaido 060-0818 Japan; 4 Department of Clinical and Population Health, College of Veterinary Medicine and Biomedical Sciences, Cavite State University, Cavite, 4122 Philippines; 5 Department of Veterinary Paraclinical Sciences, College of Veterinary Medicine, University of the Philippines Los Baños, Los Baños, Laguna, 4031 Philippines; 6 National Agriculture and Food Research Organization, 3-1-5 Kannondai, Tsukuba, Ibaraki 305-0856 Japan

## Abstract

Thogoto virus (THOV), a tick-borne arbovirus not previously reported in East Asia, was recently isolated from *Haemaphysalis longicornis* in Kyoto, Japan. In this study, we investigated the vector competence of *H. longicornis* ticks for a Japanese isolate of the Thogoto virus using anal pore microinjection and experimental virus acquisition. Our results showed that anal pore microinjection can readily infect adult ticks, and THOV-infected ticks can successfully transmit the virus to mice. Blood feeding was also critical in the distribution of the virus in tick organs, most especially in the salivary glands. Furthermore, co-feeding between an infected adult and naïve nymphs can also produce infected molted adults that can horizontally transmit THOV to mice. Altogether, our results suggest that *H. longicornis* is a competent vector for the Japanese THOV isolate and could be the primary tick vector of the virus in Japan.

## Introduction

Ticks are important vectors of viruses of public health importance, including tick-borne encephalitis virus (TBEV), Crimean-Congo hemorrhagic fever virus, and African swine fever virus, which are known to cause severe clinical symptoms in humans and domestic animals^1–3^. In Japan, the only known endemic tick-borne viruses include TBEV and severe fever with thrombocytopenia syndrome virus (SFTSV)^4,5^. However, Thogoto virus (THOV), a tick-borne virus not previously reported in East Asia, was recently isolated from a *Haemaphysalis longicornis* in Kyoto, Japan^6^. THOV is the type species of the genus Thogotovirus in the family Orthomyxoviridae^7^. THOV has a genome consisting of 6 negative-sense, single-stranded RNA segments, and it is structurally and genetically similar to influenza viruses^8,9^.

The virus was first isolated in 1960 from *Rhipicephalus* (*Boophilus*) *decoloratus* and *Rhipicephalus* spp. ticks collected on cattle in Thogoto Forest, Nairobi, Kenya^10^, and is reported to affect vertebrate hosts such as cattle, camels, and, sporadically, humans. Other ticks that are known to be vectors of this virus include *R. annulatus*, *R. sanguineus*, *R. appendiculatus*, *R. bursa*, *R. evertsi*, *Amblyomma variegatum*, *Hyalomma truncatum*, and *H. a. anatolicum*^11^. The virus can cause afebrile leucopenia in cattle and abortion in sheep^12^. THOV can also affect humans, with one fatality already recorded from two reported human cases^13^.

Since this is the first reported isolation of THOV in Japan, we are greatly interested in knowing the survival dynamics of THOV in *H. longicornis*. Moreover, it is also important to determine whether *H. longicornis* is the principal vector of THOV in Japan, since the Japanese THOV isolate was originally obtained from *H. longicorni*s, the major tick species at the collection site^6^. Ultimately, we aimed to demonstrate the vector competency of *H. longicornis* for harboring and transmitting the Japanese THOV isolate.

## Results

### THOV replication in ticks

**Figure 1:**
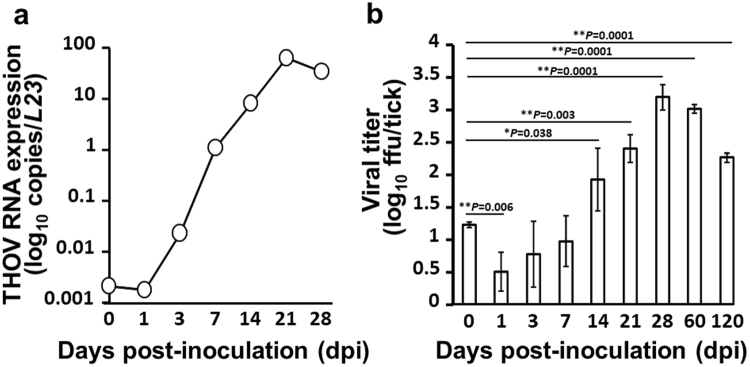
Replication of the Thogoto virus (THOV) in *Haemaphysalis longicornis* after infection using anal pore microinjection. To quantify the changes in THOV RNA collected from groups of five ticks at each time point, real-time PCR was used (**a**). Virus titration after THOV infection, wherein error bars indicate the SD in mean values of five ticks at each time point (**b**). Welch’s *t*-test: ^*^P < 0.05, ^**^P < 0.01, as compared to day 0.

In this study, we established that the introduction THOV into the tick’s gut using a microinjector can be an effective method for infecting adult ticks. Successful replication of the virus in *H. longicornis* was observed in the experimentally injected ticks. Using real-time PCR, increasing viral RNA levels can be observed beginning at 3 days post-inoculation (dpi), and eventually peaking at 21 dpi (Fig. 1a). On the other hand, Fig. 1b also shows an increasing virus titer pattern, peaking at 28 dpi. Even 4 months post-THOV inoculation, infectious virions can still be detected. We also conducted organ titration to determine which organ is the target site for virus replication in unfed and feeding states. In unfed adult ticks, THOV was consistently isolated from the midguts of infected ticks (20/20) (Table 1). On the other hand, we can also isolate infectious virions from 10% of the collected salivary glands (2/20), while no virus was isolated from the carcass (0/20). However, the percentage of infected salivary glands and carcasses increased notably during blood feeding, at 93.3% (14/15) and 100% (15/15), respectively. The midgut remains 100% (15/15) infected during blood feeding.

**Table 1:** Titration of THOV isolated from tick organs of unfed and partially fed infected ticks via focus formation assay.

Organs	Positive/Total (%)		Mean titer ± SD (log10 ffu/tick)	
	Unfed	Partially fed	Unfed	Partially fed
Midgut	20/20 (100)	15/15 (100)	2.85 ± 0.30	1.66 ± 0.57
Salivary gland	2/20 (10)	14/15 (93.3)	2.83 ± 0.29	1.38 ± 0.34
Carcass	0/20 (0)	15/15 (100)	—	1.42 ± 0.45

**Figure 2:**
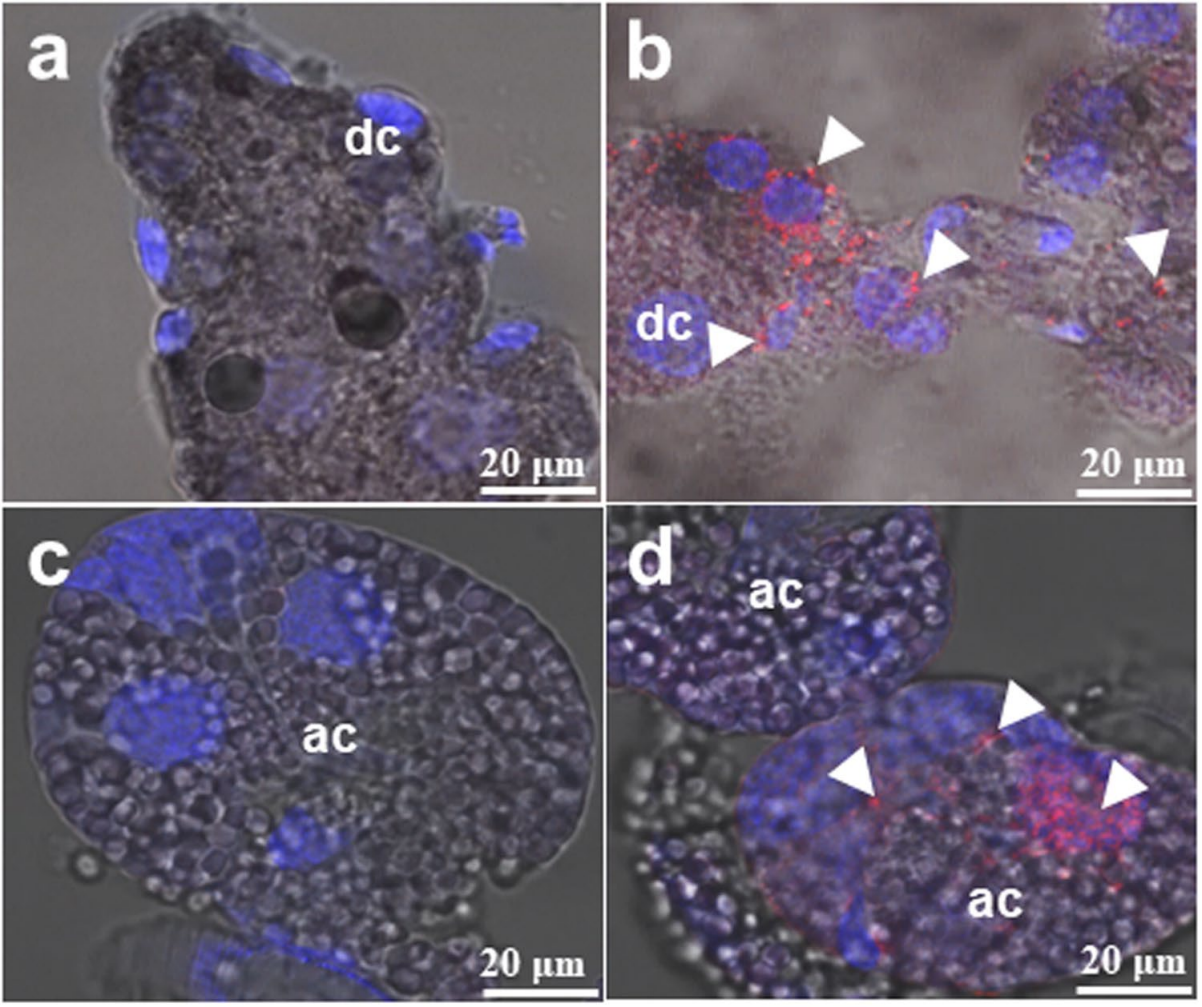
Localization of the Thogoto virus (THOV) in the midgut (**a**,**b**) and salivary glands (**c**,**d**) of unfed adult ticks after infection via anal pore microinjection. Viral antigens were detected using a specific THOV polyclonal antibody (**b**,**d**), while normal mouse serum served as a control (**a**,**c**). Nuclear counterstaining (blue) was done using DAPI, and arrowheads denote THOV antigens (red) (bar = 20 μm). dc: digestive cells; ac: acinus.

**Figure 3:**
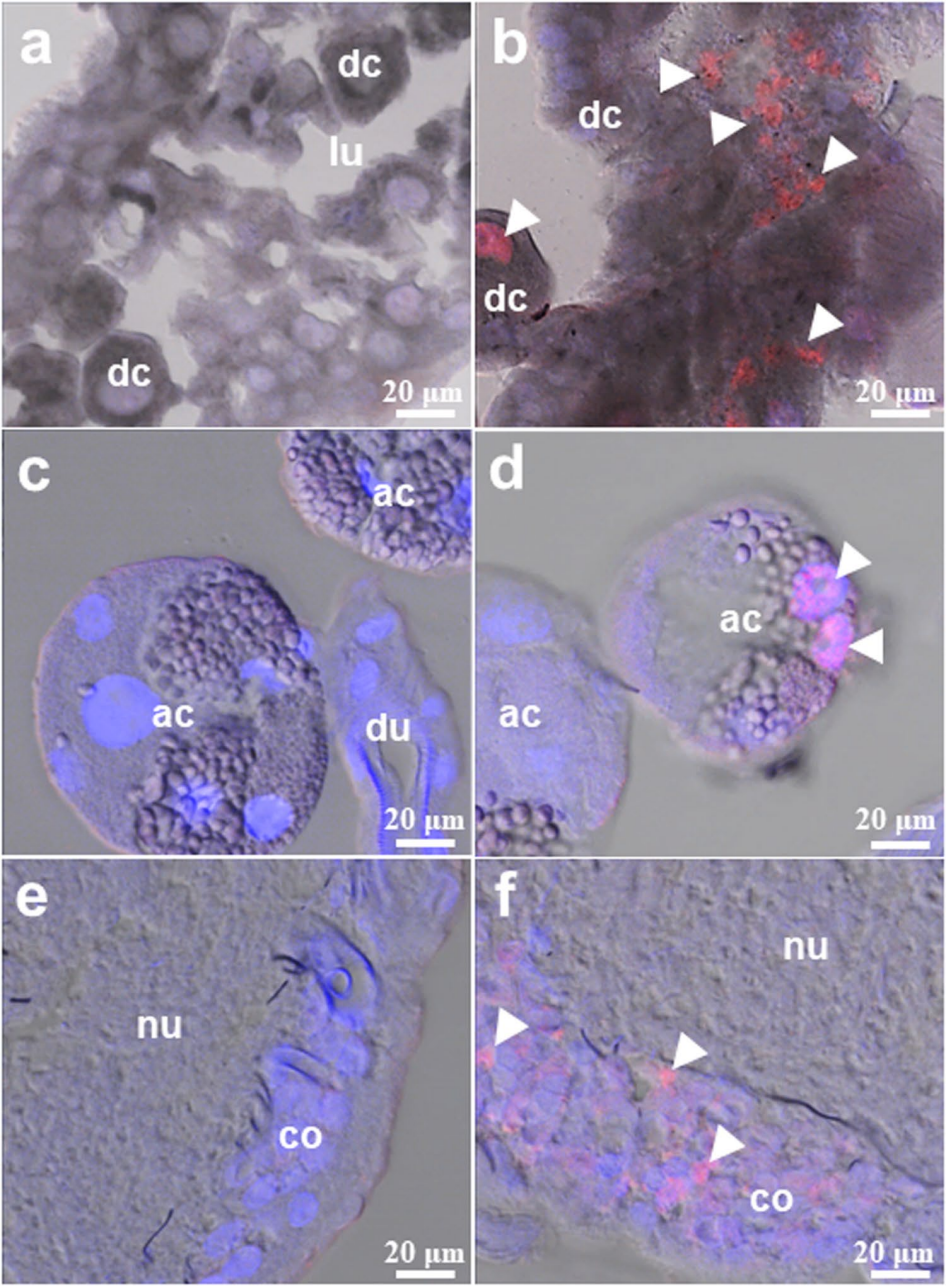
Localization of the Thogoto virus (THOV) in the midgut (**a**,**b**), salivary glands (**c**,**d**), and synganglia (**e**,**f**) of partially fed, infected adult ticks via anal pore microinjection. Viral antigens were detected using a specific THOV polyclonal antibody (**b**,**d**,**f**), while normal mouse serum served as a control (**a**,**c**,**e**). Nuclei counterstaining (blue) was done using DAPI, and arrowheads denote THOV antigens (red) (bar = 20 μm). dc: digestive cells; ac: acinus; du: duct; lu: lumen; nu: neuropile; co: cortex.

To detect viral antigens in selected organs of unfed and partially fed, adult ticks, an indirect immunofluorescent antibody test (IFAT) was used, wherein positive fluorescence denotes the presence of viral antigen. THOV was consistently detected in the midgut (Fig. 2b) and salivary glands (Fig. 2d). During blood feeding, the virus can still be detected both in the midgut (Fig. 3b) and salivary glands (Fig. 3d), although the synganglia were also notably infected (Fig. 3f).

Naïve nymphs were also evaluated to determine whether they can acquire THOV through feeding on THOV-injected mice or via co-feeding with an adult infected via anal pore microinjection. Our preliminary results on virus acquisition experiments showed that *H. longicornis* can experimentally acquire THOV from experimentally infected mouse, wherein 3.3% (1/30) of molted adult ticks from engorged nymphs fed on THOV-injected mice turned positive with THOV RNA, while 2.7% (1/36) turned positive for infectious virions (Table 2). In contrast, co-feeding between an infected adult and naïve nymphs showed much higher detection rates in the molted adults for viral RNA at 22.5% (9/40) and infectious virions at 7.9% (3/38) (Table 3).

**Table 2:** Detection and isolation of THOV from whole adult ticks that molted from nymphs fed on either EMEM- or THOV-injected mice.

Group	Viral RNA detection (%)	Virus isolation (%)
EMEM-injected mice	0/30 (0)	0/32 (0)
THOV-injected mice	1/30 (3.3)	1/36 (2.7)

**Table 3:** Detection and isolation of THOV from whole adult ticks that molted from nymphs co-fed on either EMEM- or THOV-injected adult ticks

Co-feeding group of nymphs	Viral RNA detection (%)	Virus isolation (%)
EMEM-injected adult ticks	0/20 (0)	0/45 (0)
THOV-injected adult ticks	9/40 (22.5)	3/38 (7.9)

### Tick transmission of THOV to mice

**Figure 4:**
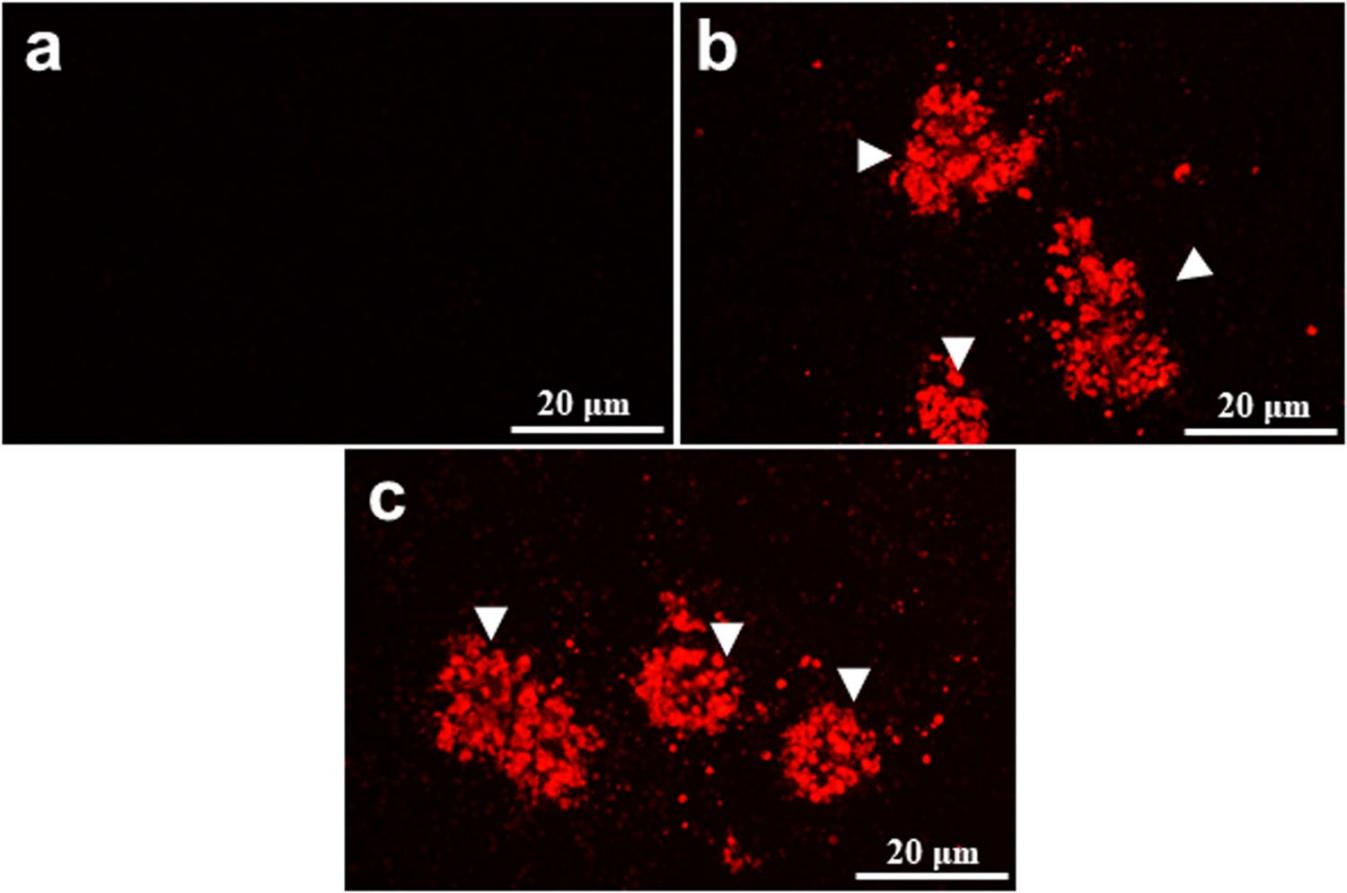
Detection of Thogoto virus (THOV) antibodies in serum samples from mice using immunofluorescence assay. Sera collected from a mouse infested with EMEM-injected ticks (**a**) (1:200, No.1); mouse inoculated with 10,000 ffu of THOV (**b**) (1:3200, No.1); mouse infested with THOV-injected ticks (**c**) (1:3200, No.6) reacting with THOV-infected baby hamster kidney BHK-21 cells. Arrowheads denote THOV ffu detected by THOV antibodies (red) (bar = 20 μm).

To establish that *H. longicornis* infected with THOV through anal pore microinjection can transmit the virus to a susceptible host, we tested the vector capacity of *H. longicornis* in transmitting THOV to mice. At the end of the virus transmission experiment (28 days), all mice were apparently healthy, and no mortality was recorded in any experimental group. As shown in Table 4, THOV-injected ticks managed to infect the infested mice, as manifested by the presence of THOV RNA in both the spleen and liver, as determined by real-time PCR. Moreover, all mice (12/12) infested with THOV-injected adult ticks also showed the presence of THOV-specific antibodies after 28 days after infestation (dai) as determined by an immunofluorescence assay (IFA). As expected, all mice (5/5) inoculated with THOV seroconverted, while those of the negative control (NC) group did not. Among the positive serum samples from mice infested with THOV-infected ticks and the positive control (PC) group, the maximum detectable IFA titer was determined to be 1:25,600. Representative IFA detection images of THOV antibodies from sera collected from all experimental groups are shown in Fig. 4.

**Table 4:** THOV transmission from ticks injected with THOV through anal pore microinjection to mice.

Treatment	Viral RNA in liver and spleen*	Seroconversion (%)(positive/total)
Medium-injected ticks	−(0/3)	0 (0/5)
THOV-infected ticks	+(10/10)	100 (12/12)
THOV-inoculated mice	+(3/3)	100 (5/5)

^*^Real-time PCR was used to detect viral RNA as represented by presence (+) or absence (−).

Since the ticks injected with THOV using anal pore microinjection were not infected via the normal tick infection route, we then alternatively utilized adult ticks that had molted from nymphs co-fed with THOV-injected ticks to also demonstrate the transmission of the virus to mice. As observed previously in our initial virus transmission experiment, no sign of disease and mortality was recorded in any experimental animal group during the entire duration of the study. However, only one infested mouse (1/18) showed THOV-specific antibodies (titer at 1:12800) at 28 dai from the THOV co-feeding group. As expected, all mice inoculated with THOV seroconverted (5/5) (maximum at titer 1:25600), while those in the EMEM co-feeding group did not (0/5).

### Detection of THOV RNA in eggs

Lastly, all fully engorged ticks collected from the tick infestation experiments (experimental anal pore microinjection and experimental virus acquisition through co-feeding) using the feeding capsule method were allowed to lay eggs. Fifty percent of the individual clutch of eggs produced from both THOV- and EMEM-injected groups was homogenized separately, while the remaining 50% of each clutch of eggs was allowed to hatch into larvae for possible future use. However, no viral RNA detection was observed from eggs collected from experimentally infected ticks via anal pore microinjection (0/22) and EMEM-injected ticks (0/8), respectively.

The same results were also observed from the co-feeding experiment, wherein no THOV RNA was detected (0/18) in eggs from collected engorged ticks. As expected, molted adult ticks from nymphs co-infested with EMEM-injected ticks did not produce infected eggs (0/5); however, these results are still preliminary as the tick infestation using adult ticks from the co-feeding experiment was only conducted once.

## Discussion

In this study, anal pore microinjection proved to be an effective method for infecting ticks with THOV with no apparent mortality. As shown in Fig. 1, THOV can successfully infect and replicate in *H. longicornis* as observed in increasing levels of THOV RNA and virus titers.

On the other hand, IFAT results show that THOV mainly localizes in the midgut and salivary glands and, additionally, in the synganglia during feeding. However, organ titration revealed that digestive cells could be the primary replication site of the virus, since THOV was only consistently isolated from the midgut, especially in the unfed state. Such an observation is crucial, since one of the most important determinants of vector competence is the susceptibility of midgut cells to virus infection^2^.

However, blood feeding greatly influenced the distribution of the virus in tick organs, most especially in the salivary gland. In the unfed state, only 10% of salivary glands were positive for infectious virions, as compared to 93.3% in partially fed ticks. These observations show that the virus readily transfers from the midgut to the salivary gland during feeding to facilitate the transmission of the virus to the host. For most tick-borne pathogens present in the tick gut, dissemination into the hemolymph and migration to the salivary glands can happen immediately after acquisition or after the stimulus of a new blood meal^14^. Such a phenomenon was previously reported for TBEV, wherein feeding enhances salivary gland infection; thus, partially fed ticks have significantly higher infection prevalences than unfed ticks from the same collection site^15^.

Infected synganglia, which could have also been infected from the virus present in the hemolymph during blood feeding, are consistent with the tropism of the virus in the organ. Synganglion infection by tick-borne viruses has already been reported previously, especially for THOV in *R. appendiculatus*^16^ and for Langat virus and Powassan virus in *Ixodes scapularis*^17^.

Virus transmission experiments also showed that *H. longicornis* is a competent vector of THOV, as virus-injected ticks managed to infect the infested mice, as manifested by THOV RNA expression in both the spleen and liver and THOV-specific antibodies, but with no sign of disease and mortality for the whole duration of the study. Likewise, the absence of clinical signs and deaths in the present results still remains consistent with the previous results indicating that THOV-Kamigamo strain has a low-pathogenic characteristic^6^, as previously shown by the absence of mortality in the challenged groups.

The current study also managed to demonstrate that naïve nymphs can be infected by THOV through natural routes of infections. Despite the small number of ticks examined, our preliminary results on the infection of nymphs through blood feeding on THOV-injected mice, proved to be possible at 3.3% in the present study; however, co-feeding the naïve nymphs with an infected adult showed a higher infection rate, at 22.5%. This occurrence was previously reported for THOV that was transmitted more efficiently by *R. appendiculatus* via non-viraemic guinea pigs (co-feeding between infected and uninfected ticks) than via highly viraemic hamsters^18^. The transmission between co-feeding infected and uninfected ticks without systemic infection was also demonstrated in other tick-borne viruses, such as the Crimean-Congo hemorrhagic fever virus, Kyasanur Forest Disease virus, Louping ill virus, and TBEV^19^. Transstadial maintenance of THOV in *H. longicornis* was also established in the present study, since the engorged nymphs produced infected adults, suggesting that the virus can survive the harsh molting process. The non-detection of THOV in eggs in the present study suggests that no vertical transmission occurred, although more samples are needed to fully support this observation. In contrast, the infection of nymphal ticks through co-feeding with infected adults was clearly established. It was previously reported that in the presence of weak vertical transmission, the infection of nymphal ticks plays an important role in the virus transmission cycle that is critical in pathogen maintenance^20^. On the other hand, as compared to the experimentally infected ticks, the group of transstadially infected adults showed a considerably lower transmission rate as compared to that of experimentally infected adults.

In conclusion, our findings indicate that *H. longicornis*, a widely distributed tick in Japan, is a competent vector of the Japanese THOV isolate. However, additional studies are needed to identify which animals potentially serve as the reservoir host of the virus and to elucidate animal susceptibility and the geographic distribution of THOV infections in Japan.

## Methods

### Ticks and animals

Parthenogenetic *H. longicornis* (Okayama strain) ticks were maintained for several generations by feeding on the ears of Japanese white rabbits (*KBT* Oriental Co., Saga, Japan) at the Experimental Animal Center, Joint Faculty of Veterinary Medicine, Kagoshima University, Kagoshima, Japan. Conversely, ticks were infested on 6-week-old female BALB/c mice (Kyudo, Fukuoka, Japan) using the feeding capsule method as previously described^21^. Animal experiments were conducted in accordance with approved guidelines (approval numbers VM 15005 and VM 16016) of the Animal Care and Use Committee of Kagoshima University.

### Cell culture and virus

Baby hamster kidney (BHK-21) cells were grown in Eagle’s Minimum Essential Medium (EMEM) containing 5% fetal bovine serum (FBS) and 1% antibiotic/antimycotic and maintained at 37 °C under 5% CO_2_ until use. The THOV Kamigamo strain was amplified in BHK-21 cells, and the virus stock titer was determined by focus formation assay (FFA) as previously described with some modifications^22^. Briefly, BHK-21 cells (1 × 10^5^ cells/well) were plated in 24-well plates. Then 10-fold serial dilutions of the virus stock were added on each well for 1 h at 37 °C. Unabsorbed viruses were removed by rinsing cells with sterile PBS at least once. The infected cells were overlaid with 1.5% methylcellulose containing Modified Eagle’s Medium (MEM) (Gibco, California, USA) with 1% FBS and 1% antibiotic/antimycotic. The viral foci were detected by a primary antibody (hyperimmune mouse polyclonal IgG) against THOV surface proteins followed by Alexa Fluor® 594 goat anti-mouse IgG (Invitrogen, California, USA) 3–4 days post infection.

The number of foci was counted using a fluorescence microscope mounted with a DP71 camera (Olympus Corporation, Tokyo, Japan) and the titer of virus stock was expressed as focus forming unit (ffu). The virus stock was later aliquoted and stored at −80 °C.

### Infection of ticks with THOV

Adult ticks were infected experimentally with THOV by anal pore microinjection as previously described^23,24^. Infection by microinjection was accomplished by injecting 0.3 µl of virus stock containing approximately 100 ffu of THOV into the tick’s anal aperture using a microinjector (Narishige Group, Tokyo, Japan). In contrast, EMEM was injected in the control group. After the injection, ticks were kept for 24 h in a 25 °C incubator to observe for any mortality arising from possible injury due to the injection.

Alternatively, naïve nymphs were also evaluated to determine whether they can acquire THOV through feeding on THOV-injected mice or via co-feeding with an adult infected via anal pore microinjection. For feeding in THOV-injected mice, 3 mice were injected subcutaneously with 10,000 ffu of THOV, and then immediately infested with 30 naïve nymphs each. Three EMEM-injected mice, which served as the negative controls, were infested with the same number of nymphal ticks per mice. For the co-feeding experiment, 5 THOV-infected adult ticks (28 days post-inoculation) were infested individually onto 5 separate mice together with 20 naïve nymphs/mouse. Four EMEM-injected adult ticks served as negative controls with the same number of nymphs/mouse. After feeding, all engorged nymphs were collected and allowed to molt. Twenty-one days post-molting, adult ticks were examined for either the presence of THOV RNA or infectious virions via real-time PCR and focus formation assay, respectively.

### Detection of THOV RNA in mouse and tick tissues

Total mRNA extraction and analysis of THOV RNA in mice and ticks through real-time PCR using THUNDERBIRD™ SYBR^®^ qPCR Mix (TOYOBO, Osaka, Japan) with a 7300 real-time PCR system (Applied Biosystems, California, USA) were performed as previously described^25^. Briefly, gene-specific primers were designed to target THOV segment 6 (matrix protein) and tick ribosomal protein *L23* or mouse *β-actin* (internal control) genes (Table 5). To generate standard curves, eightfold serial dilutions of the cDNA from THOV, adult ticks, or mouse tissue were used. Each sample was run in triplicate, and the data were analyzed using 7300 System SDS software (Applied Biosystems). Lastly, normalized gene expressions were computed by dividing the amount of *THOV* gene expression by the amount of *L23* or mouse *β-actin* expressions for each sample.

**Table 5:** List of real-time PCR primers used for the detection of THOV in ticks and mice.

Primer name	Primer sequence (5′-3′)
THOV Forward	CGGATGGCAACAAGAAGCTG
THOV Reverse	AATCAGCACAACATCCCGGT
Mouse β-Actin F	TTCTTTGCAGCTCCTTCGTT
Mouse β-Actin Reverse	ATGGAGGGGAATACAGCCC
*H. longicornis* L23 Forward	CACACTCGTGTTCATCGTCC
*H. longicornis* L23 Reverse	ATGAGTGTGTTCACGTTGGC

### Isolation and titration of THOV from tick tissues and whole adult ticks

Ticks inoculated with THOV were collected and individually homogenized at 0, 1, 3, 7, 14, 21, 28, 60, and 120 dpi. The collected individual homogenate was eventually titrated as previously described^22^.

On the other hand, collected tick organs such as salivary glands, midguts, and carcasses (without midguts and salivary glands) were washed 3 times with PBS, homogenized individually, and then diluted with 300 µl of E-MEM with antibiotics, centrifuged, and, subsequently, filtered as previously described^24^. The collected supernatants were directly used for titration.

### Detection of THOV antigens in tick organs using IFAT

IFAT was performed to demonstrate the localization of THOV in salivary glands and midguts of unfed, THOV-injected ticks, while, for partially fed ticks, synganglia were also used, following the method described previously^26,27^.

### Tick transmission of THOV to mice

To determine whether THOV introduced into ticks via anal pore microinjection can be transmitted to mice by tick bite, we allowed THOV-infected adults to feed on mice via the feeding capsule/tube method^21^. Twenty-two infected adult ticks (28 dpi) were allowed to feed individually on 22 naive mice until engorgement. EMEM-injected ticks were also allowed to feed on 8 mice to serve as NC, while another 8 mice were injected with 10,000 ffu of THOV to serve as PC. Seven dai, spleen and liver tissues were obtained from 10 mice infested with THOV-injected ticks and examined by real-time PCR to detect THOV RNA as described above. Three NC and PC mice were also sacrificed for spleen and liver collection. Then, the remaining mice from each group were observed for up to 28 dai. At the end of the observation period (28 dai), blood samples were collected for the detection of THOV-specific antibodies from the different experimental groups using IFA^28^. The result shown is representative of two experiments with the same result. Briefly, serum samples were assayed by using THOV-infected BHK cells as antigens. The cells were cultivated in 48-well plates, fixed with 4% paraformaldehyde, and used as antigens for IFAs to detect THOV antibodies in the collected serum samples. Each serum was initially assayed at a 1:200 dilution (5% skimmed milk in PBS), and then a twofold diluted, thereafter.

Alternatively, we also allowed the molted adult ticks from nymphs co-infested with THOV-infected adults via anal pore microinjection to feed on mice. Twenty (20) molted adult ticks (28 days post molting) were allowed to feed individually on 20 naive mice until engorgement via the feeding capsule/tube method. EMEM-injected ticks were also allowed to feed on 5 mice to serve as NC, while another 5 mice were injected with 10,000 ffu of THOV to serve as PC. At 28 dai, blood samples were collected for the detection of THOV-specific antibodies from the different experimental groups using IFA as described above. This experiment was only conducted once.

### Determination of transovarial transmission of THOV in ticks

All fully engorged ticks collected from the tick infestation experiments (experimental anal pore microinjection and experimental virus acquisition through co-feeding) using the feeding capsule method were allowed to lay eggs. Fifty percent of the individual clutch of eggs produced from both THOV- and EMEM-injected groups was homogenized separately, while the remaining 50% of each clutch of eggs was allowed to hatch into larvae and, later, homogenized 14 days after hatching. Both egg and larval homogenates were used to isolate total RNA for cDNA synthesis. THOV RNA was detected in each sample using real-time PCR as described above.

### Statistical analysis

All samples were tested at least in triplicate unless otherwise stated and statistically analysed using Welch’s *t-*test (non-parametric, two-tailed) in GraphPad Prism version 3.0 (GraphPad Software, Inc., CA, USA), wherein *P* values of less than 0.05 and 0.01 were regarded as significant and highly significant, respectively.
